# The Role of Lactate Metabolism in Prostate Cancer Progression and Metastases Revealed by Dual-Agent Hyperpolarized ^13^C MRSI

**DOI:** 10.3390/cancers11020257

**Published:** 2019-02-22

**Authors:** Robert Bok, Jessie Lee, Renuka Sriram, Kayvan Keshari, Subramaniam Sukumar, Saeed Daneshmandi, David E. Korenchan, Robert R. Flavell, Daniel B. Vigneron, John Kurhanewicz, Pankaj Seth

**Affiliations:** 1 Department of Radiology and Biomedical Imaging, University of California, San Francisco, CA 94143, USA; Robert.Bok@ucsf.edu (R.B.); Jessie.Lee@ucsf.edu (J.L.); Renuka.Sriram@ucsf.edu (R.S.); ssukumar@live.com (S.S.); Dave.Korenchan@ucsf.edu (D.E.K.); Robert.Flavell@ucsf.edu (R.R.F.); Dan.Vigneron@ucsf.edu (D.B.V.); 2 Department of Radiology, Memorial Sloan-Kettering Cancer Center (MSKCC), New York, NY 10065, USA; rahimikk@mskcc.org; 3 Molecular Pharmacology Program, Memorial Sloan Kettering Cancer Center, New York, NY 10065, USA; 4 Department of Radiology, Weill Cornell Medical College, New York, NY 10065, USA; 5 Department of Medicine, Division of Interdisciplinary Medicine, Beth Israel Deaconess Medical Center, Beth Israel Cancer Center, Harvard Medical School, Boston, MA 02215, USA; sdaneshm@bidmc.harvard.edu

**Keywords:** hyperpolarized ^13^C, prostate cancer, lactate, magnetic resonance imaging, lactate dehydrogenase

## Abstract

This study applied a dual-agent, ^13^C-pyruvate and ^13^C-urea, hyperpolarized ^13^C magnetic resonance spectroscopic imaging (MRSI) and multi-parametric (mp) ^1^H magnetic resonance imaging (MRI) approach in the transgenic adenocarcinoma of mouse prostate (TRAMP) model to investigate changes in tumor perfusion and lactate metabolism during prostate cancer development, progression and metastases, and after lactate dehydrogenase-A (LDHA) knock-out. An increased Warburg effect, as measured by an elevated hyperpolarized (HP) Lactate/Pyruvate (Lac/Pyr) ratio, and associated *Ldha* expression and LDH activity were significantly higher in high- versus low-grade TRAMP tumors and normal prostates. The hypoxic tumor microenvironment in high-grade tumors, as measured by significantly decreased HP ^13^C-urea perfusion and increased PIM staining, played a key role in increasing lactate production through increased *Hif1α* and then *Ldha* expression. Increased lactate induced *Mct4* expression and an acidic tumor microenvironment that provided a potential mechanism for the observed high rate of lymph node (86%) and liver (33%) metastases. The *Ldha* knockdown in the triple-transgenic mouse model of prostate cancer resulted in a significant reduction in HP Lac/Pyr, which preceded a reduction in tumor volume or apparent water diffusion coefficient (ADC). The *Ldha* gene knockdown significantly reduced primary tumor growth and reduced lymph node and visceral metastases. These data suggested a metabolic transformation from low- to high-grade prostate cancer including an increased Warburg effect, decreased perfusion, and increased metastatic potential. Moreover, these data suggested that LDH activity and lactate are required for tumor progression. The lactate metabolism changes during prostate cancer provided the motivation for applying hyperpolarized ^13^C MRSI to detect aggressive disease at diagnosis and predict early therapeutic response.

## 1. Introduction

Although a majority of newly diagnosed prostate cancer patients (164,690 men) will have indolent, non-life threatening disease, an estimated 29,430 men died of metastatic prostate cancer in the United States alone in 2018 [1]. While those with indolent tumors can be managed with active surveillance, approximately 30% of these patients will be reclassified as higher risk for disease progression, requiring definitive therapy [2,3]. There is an unmet clinical need for an accurate, non-invasive imaging method to detect aggressive, clinically significant cancer early in these patients so timely treatment of this potentially deadly disease can be initiated. There is growing evidence that the ability of cancer cells to invade adjacent normal tissues and locally grow and metastasize to distant sites is significantly impacted by changes in tumor cellular metabolism and environmental conditions such as reduced perfusion and hypoxia [4]. Specifically, it has been hypothesized that the Warburg effect, an up-regulation of aerobic glycolysis and production of lactate, the result of the lactate dehydrogenase (LDH) reaction, is an adaptation of cancer cells that aids in survival, growth, and metastasis [4]. Lactate dehydrogenase-A (LDHA), a protein subunit of the highly lactate-favoring LDH isoform muscle-type 5 (LDH-5), catalyzes the reduction of pyruvate to lactate and is overexpressed in many cancers, including prostate tumors [5,6]. Aerobic glycolysis is increased in prostate cancer due in part to genomic loss of the *PTEN* (*phosphatase and tensin homolog*) locus, leading to activation of the PI3K/AKT pathway, and 8q amplification, including of the *Myc* gene, which occurs in up to 70% and 30% of prostate cancers, respectively [7]. Also, tumor microenvironment factors, such as reduced perfusion and hypoxia can further increase aerobic glycolysis [4]. The oxygen-sensitive Hif-1α transcription factor is up-regulated in regions of tumor hypoxia and increases aerobic glycolysis by increasing the expression and activity of key enzymes in the glycolytic pathway, such as LDHA as well as monocarboxylate transporters (MCT1 and 4) responsible for the transport of pyruvate and lactate in and out of the cell [8,9].

Tumor cell export of lactic and other acids, combined with poor tumor perfusion, results in an acidic extracellular pH in tumors compared with normal tissue under physiologic conditions [10]. The resulting acidic environment promotes cancer aggressiveness and metastasis by facilitating a degradation of the extracellular matrix by proteinases [11,12], increasing angiogenesis through the release of VEGF (vascular endothelial growth factor) [13], and inhibiting the immune response to tumor antigens [14]. Taken together, these observations suggest that interventions to reverse the Warburg effect, such as the inhibition of LDHA, may harm cancer cells by depriving them of these survival mechanisms [15,16]. Normal cells should be largely unharmed by such therapies as they have a much lower reliance on the Warburg effect. In this study, we applied a metabolic imaging approach with a new dual-agent hyperpolarized (HP) ^13^C magnetic resonance imaging (MRI) exam to investigate the interplay between prostate cancer metabolism and the microenvironment and to elucidate the functional role of LDHA in prostate cancer progression and metastases using the transgenic adenocarcinoma of mouse prostate (TRAMP) model.

The TRAMP is a transgenic animal model in which tumor development is targeted specifically to the murine prostate as a consequence of the overexpression of the SV40 T antigen. Histologically, TRAMP mice develop a prostatic intraepithelial neoplasia (PIN) by 8–12 weeks of age that progresses to adenocarcinoma with distant metastases (predominately lymph node metastases) by 24–30 weeks of age. Tumors progress from androgen dependence to independence, and essentially all males develop tumors [17]. Of particular importance to these studies is that changes in TRAMP prostate and tumor metabolism and progression mimic the human disease [9,18]. Moreover, we have generated and utilized in this study an inducible cre-lox mouse model of inducible *Ldha* knock-out (Cre^tm^-LDHA^fl/fl^; TRAMP) to study the consequences of inhibiting the Warburg effect on prostate cancer metabolism, progression and metastases for the first time.

Hyperpolarized ^13^C magnetic resonance spectroscopic imaging (MRSI) is a powerful new metabolic imaging method which uses specialized instrumentation to provide signal enhancements of over 10,000-fold for ^13^C magnetic resonance imaging and spectroscopy using enriched, safe, endogenous, non-radioactive compounds [18]. While prostate cancer is often inadequately evaluated using FDG-PET (fluorodeoxyglucose-positron emission tomography; which assesses glucose uptake and phosphorylation [19,20]), the HP ^13^C MRSI detects down-stream metabolism, specifically the metabolic flux of HP ^13^C-pyruvate to lactate catalyzed by lactate dehydrogenase. Another important feature of the HP ^13^C MRSI is that it encodes chemical as well as spatial information, thereby providing the potential for using multiple hyperpolarized ^13^C-labeled probes to detect several metabolic and/or physiologic processes simultaneously after the injection of a single bolus [21]. HP ^13^C-urea is not taken up and metabolized by most mammalian tissues and prior publications have demonstrated that hyperpolarized ^13^C-urea provides an assessment of tumor perfusion in animal cancer models [21,22]. Methods for co-polarizing ^13^C-pyruvate and ^13^C-urea have been developed, and the two agents successfully polarized, and injected in pre-clinical models to simultaneously measure perfusion and metabolism [21,22].

The goal of this study was to use a dual-agent, ^13^C-pyruvate and ^13^C-urea, HP ^13^C MRSI approach to investigate changes in tumor perfusion and metabolism during prostate cancer development, progression from low- to high-grade disease and metastases, and after *Ldha* knock-out in order to assess the functional importance of lactate metabolism in this common and often lethal cancer.

## 2. Materials and Methods

### 2.1. Animal Protocol and Handling

All animal studies were conducted in accordance with the policies of the Institutional Animal Care and Use Committee (IACUC) at the University of California, San Francisco (UCSF) (AN170092-01B, approved on 11 September 2017) and Beth Israel Deaconess Medical Center (BIDMC) (040-2016, approved on 18 August 2018). The transgenic mouse model of prostate cancer (TRAMP) was supplied by Roswell Park Cancer Institute (Buffalo, NY, USA). The TRAMP mice utilized in the prostate cancer development and progression portions of this study were generated in the C57BL6/FVB strain background and prepared as previously described [17], and were 17–36 weeks of age. These TRAMP mice were supplied by Roswell Park Cancer Institute (Buffalo, NY, USA). In the TRAMP model there is not a specific time or age at which individual mice develop tumors nor a specific time or age at which tumors progress from low to high grade. In this respect, the heterogeneity in the TRAMP model mimics what occurs in the human disease. Therefore, mice were screened using ^1^H MRI to select mice with normal prostates and low- and high-grade disease for subsequent study. To study metabolic and perfusion changes tumor perfusion during prostate cancer development and progression, we performed ^1^H (T_2_ wt. anatomic imaging and diffusion-weighted) MRI and HP ^13^C MRSI (HP ^13^C-pyruvate and ^13^C-urea) on normal mouse prostates (*n* = 4), low-grade TRAMP tumors (*n* = 9) and high-grade TRAMP tumors (*n* = 11) prior to euthanization and subsequent pathologic, immunohistochemistry (IHC) and mRNA expression and enzyme activity analyses. Tumor grade was determined as described in a later section.

**Figure 1:**
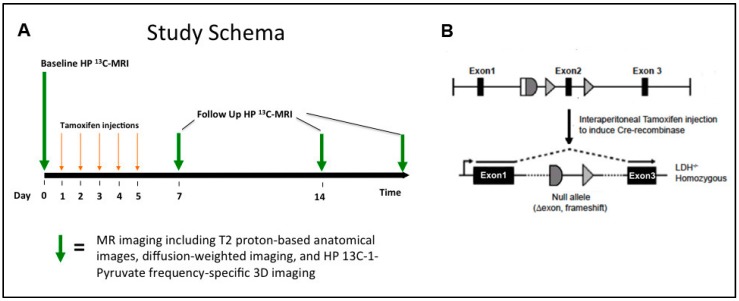
(**A**) Schematic of the study design for lactate dehydrogenase-A (LDHA) knockout studies. Multi-parametric ^1^H and HP ^13^C magnetic resonance imaging (MRI) exams performed pre- and post-LDHA knockdown. (**B**) Schematic of LDHA gene knockdown induced by tamoxifen administration.

Triple transgenic mice bearing the oncogenic T-antigens under the control of the rodent probasin promoter, the cre recombinase, and floxed murine *Ldha* genes were generated by crossing TRAMP mice in the C57BL/6 strain background with Cre^tm^-LDHA^fl/fl^ mice (Figure 1B, left). Administration of intraperitoneal tamoxifen allowed temporal control of the deletion of the LDHA gene. We utilized this genetically engineered model to carry out HP ^13^C MRSI studies according to the schema outlined in Figure 1A. Baseline TRAMP tumors were selected based on evidence of an adequately sized tumor for follow-up ^1^H/HP ^13^C MR imaging studies based on weekly ^1^H MRI screening exams. The volumes of TRAMP tumors used in this study should have reflected a combination of low- and high-grade disease. However, since this was a longitudinal study in which tumor bearing TRAMP mice went on for *Ldha*-knockout, a pathologic assessment of low- and high-grade tumors at baseline was not possible. Subsequently, a baseline ^1^H/HP ^13^C MR imaging study was performed, in which T_2_-weighted, diffusion-weighted, and HP ^13^C MRSI (HP [1-^13^C] pyruvate and [^13^C] urea) data were collected. Mice (*n* = 17) were then administered a 4–5-day course of tamoxifen (80–100 μL of 33.3 mg/mL solution) and MRI studies were repeated at approximately 1-week intervals for 2–3 weeks. There was a 90% knockdown of LDHA by western blot analyses in the TRAMP mouse by 7 days after administration (Figure S1). Control animals (*n* = 8) were administered vehicle only (corn oil without tamoxifen). After imaging mice were euthanized and subsequent pathological, histopathological, IHC and mRNA expression and enzyme activity analyses were performed.

### 2.2. Hyperpolarization of ^13^C-Labelled Compounds

A HyperSense™ DNP polarizer (Oxford Instruments, Abingdon, UK) was used to polarize the ^13^C probes as described previously [21]. Twenty-four micro liters of neat [1-^13^C] pyruvic acid (Isotec Stable Isotopes, Miamisburg, OH, USA) with 16.5 mM trityl radical [tris(8-carboxy-2,2,6,6,-tetra(methoxyethyl)benzo[1,2-d:4,5-d’]bis(1,3)dithiole-4-yl)methyl sodium salt] (GE healthcare, Waukesha, WI, USA) and 1.5 mM Dotarem^®^ (Guerbet) and 55 µL [^13^C] urea (6.4 M in glycerol, (Isotec Stable Isotopes, Miamisburg, OH, USA) with 17.5 mM trityl radical OX63 (Oxford Instruments) and 0.2 mM Dotarem^®^ were co-polarized. Optimum polarization was achieved by adding the urea and pyruvic acid solutions to a sample cup separately and freezing them rapidly in a liquid nitrogen bath to form two separate glass layers, as previously described [21]. This was followed by dissolution in 4.5 mL of buffer containing 40 mM Tris, 80 mM NaOH, and 0.3 mM Na_2_EDTA. The resulting dissolution mixture contained 80 mM [1-^13^C] pyruvic acid and 74 mM [^13^C] urea with average polarizations of 24 ± 5% and 18 ± 6%, respectively, and an average pH of 7.0 ± 0.5.

### 2.3. ^1^H/HP ^13^C MR Imaging Studies

All MRI experiments were done using a Varian 14.1T imaging spectrometer with a 98 mm bore vertical magnet and controlled by a direct-drive console (Agilent Technologies, Santa Clara, CA, USA). The system was equipped with 55 mm 100 G/cm gradients, a quadrature 40 mm proton coil (Agilent Inc) used for obtaining proton images, and a 40 mm diameter proton/carbon dual tuned RF (radiofrequency) coil (M2M Imaging) used for HP ^13^C MRSI in the same imaging session without altering the animal position. At the time of the MRI experiment, the mice were cannulated using a 32-gauge IV catheter in the lateral tail vein and anesthetized with 1–1.5% isoflurane/100% oxygen at a rate of 1 L/min on a heated water bed to maintain physiological body temperature and positioned vertically in the magnet using a custom animal positioning apparatus. Their respiration was continuously monitored using an animal monitoring system (SA Instruments, Stony Brook, NY, USA). For ^1^H MRI studies, data acquisition was triggered with respiration to reduce motion artifacts. For the tumor development and progression studies, ^1^H/HP ^13^C MRSI studies were performed at a single time-point, while for the *Ldha*-knockout, the imaging experiments were performed at baseline and, following the administration of tamoxifen or vehicle (corn oil), repeated at ~1-week intervals for 2 to 3 weeks. TRAMP mice were humanely euthanized and dissected and tissues harvested within 6 h of the MRI study. To measure tumor hypoxia, Pimonidazole (PIM; Hypoxyprobe^TM^, Burlington, MA, USA) solution was injected approximately 45 min prior to euthanasia at 60 mg/kg.

#### 2.3.1. ^1^H MRI

Respiratory-gated T_2_-weighted ^1^H anatomical images were acquired using a spin-echo pulse sequence (Axial: FOV = 40 × 40 mm, 256 × 256 matrix, slice thickness = 1 mm with 0.25 mm gap), with a total of 24 slices covering the entire tumor with a repetition time of 1.6 s and an echo time of 20 ms resulting in a total acquisition time of ≈7 min. Diffusion weighted imaging (DWI) was performed using a spin-echo sequence with the diffusion-sensitizing gradient applied along the y-axis; and a TE/TR = 20/1600 ms, matrix = 128 × 128 zero-filled to 256 × 256, FOV = 40 × 40 mm^2^, slice thickness = 1 mm with 0.25 mm gap, 18 slices, gradient duration = 2 ms, delay between gradients = 13 ms, and b values of with b-values of 25, 180, 323, 508 s/mm^2^). Water apparent diffusion coefficient (ADC) maps were calculated using the monoexponential function ($\frac{S\left(b\right)}{S_{0}}=e^{\left(-b\times ADC\right)}$) in VNMRJ 3.1A software (Varian, Inc., Palo Alto, CA, USA).

#### 2.3.2. HP ^13^C MRSI

**Figure 2:**
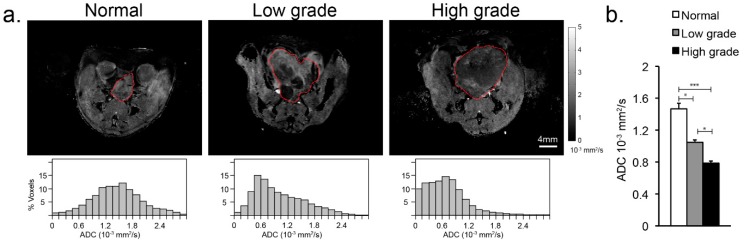
(**a**) (top) Representative apparent water diffusion coefficient images (ADC, outlined in red) overlaid on corresponding T_2_-weighted anatomic images of the normal murine prostate, low- and high-grade prostate cancer. (**a**) (bottom) Plots of the distribution of water ADC values across the normal murine prostate and low- and high-grade TRAMP tumors. (**b**) Bar graph of the mean ± sdev ADC values for normal prostate, high- and low-grade cancer. * denotes *p* < 0.05, *** for *p* < 0.0005.

Single time-point, frequency-specific ^13^C 3D imaging was performed 35 s after an injection of co-polarized [1-^13^C] pyruvate and ^13^C-urea was performed on the mice using a gradient spin-echo (GRASE) sequence [23] with multiple 180° refocusing pulses during the echo train to minimize T_2_* (*: off-frequency transverse spin relaxation) effects. The 35 s delay was based on when maximum hyperpolarized lactate production was observed using dynamic slab-selective ^13^C spectroscopy. The 90° and 180° pulses used in the sequence were chemical shift selective pulses (6 ms SLR pulse) designed to excite only resonances of interest. Resonances of [1-^13^C] lactate, [1-^13^C] alanine, [1-^13^C] pyruvate, and [^13^C] urea were excited and imaged sequentially (Figure 2). Phase encoded steps were kept at 16 × 12 × 12 and a 40 × 40 × 40 mm^3^ FOV, 2.5 × 3.3 × 3.3 mm^3^ resolution and scan time of 153 ms per image. The ^13^C data were zero-filled to 32 × 32 × 32 and the magnitude images were reconstructed to a 1.25 × 1.25 × 1.25 mm^3^ resolution. ^13^C data were acquired in the following order: (1) a 2-degree flip angle slab-selective spectrum, (2) the 3D images of pyruvate, lactate, and urea using a 3D GRASE sequence and a 90-degree flip angle spectrum. The spectra served as a supplemental verification of proper polarization levels, sufficient spectral resolution of the HP ^13^C labeled metabolites, and successful injections.

### 2.4. Histopathologic Analysis

An experienced murine pathologist performed dissection after reviewing the HP ^13^C data and the T_2_-weighted anatomical images. During the dissection process, digital images were taken as a reference for localization and registration of tumor specimens. The excised tissue was aliquoted for histochemical processing, gene expression and activity analyses. Formalin-fixed, paraffin-embedded tissue sections were stained by H&E (hematoxylin and eosin) or immunohistochemically with anti-Ki-67 (DAKO, Carpinteria CA, USA) or anti-PIM (HP2-200, Hypoxyprobe, HPI, Burlington MA, USA) antibodies. For primary tumors, the histological index, as defined in Albers et al., was used to pathologically define high- versus low-grade TRAMP tumors in this study [24]. The histologic index was calculated based on the weighted percentages of tumor differentiation (normal, well-differentiated, moderately well differentiated and poorly differentiated) from standard H&E staining. The histologic index ranged between 0 and 3, where 0 indicated that 100% of the tissue was normal and 3 indicated that 100% of the tissue was poorly differentiated; low-grade tumors had an index <2 and high-grade had an index ≥ 2.

The prevalence of metastases in control TRAMP (*Ldha*-intact) and in *Ldha*-knockout TRAMP, was calculated by assessing individual animals for either the presence or absence of metastatic disease in regional periaortic lymph nodes (PALN), more distant perirenal lymph nodes (PRLN), and visceral metastases (liver and lung), via close visual inspection and photography, at the time of dissection.

### 2.5. mRNA Expression and Enzyme Activity Analyses

Total RNA was extracted and purified from freshly frozen TRAMP tumor tissues using RNeasy Kits (Qiagen, Limburg, Netherlands). Total RNA was measured using an RNA6000 Kit (Agilent, Santa Clara, CA, USA). The RNA extract was then subjected to RQ1 RNAse-free DNAse (Promega Manufacturing, Madison, WI, USA) to digest in-sample gDNA. cDNA was synthesized by reverse transcription using a qScript DNA Synthesis Kit (Quanta Biosciences, Gaithersburg, MD, USA). Quantitative qRT-PCR was then performed to acquire the threshold cycle C_T_ value (ABI 7900HT, Applied Biosystems, Carlsbad, CA, USA). Primers (TaqMan, Life Technologies, Carlsbad, CA, USA) were selected for quantitative amplification of the genes: hypoxia-inducible factor *Hif1α*, lactate dehydrogenase subunits (*Ldha*, *Ldhb*), monocarboxylate transporters (*Mct1*, *Mct4*) and vascular endothelial growth factor (*Vegf*) relative to the housekeeping gene hypoxanthine-guanine phosphoribosyltransferase (*Hprt*).

LDH activity was measured spectrophotometrically (Infinite M200, Tecan, Switzerland) as described previously [25]. Homogenized tumor tissue incubated with serial dilutions of pyruvate and excess co-factors were measured at 340 nm to observe the linear decrease in absorbance due to NADH oxidation. The maximum velocity (Vm) and Michaelis–Menten constant (Km) and Vmax were calculated, and the activity was normalized to total protein concentration (Quick Start Bradford Protein Assay, Bio-Rad, Hercules, CA, USA).

### 2.6. Data Analyses

Imaging data analyses were performed using custom software employing the commercial IDL (Exelis Visual Information Solutions, Inc., Boulder, CO, USA) and MATLAB (Mathworks, Nattick, MA, USA) platforms, and the data was displayed using the open-source SIVIC package [26]. Tumor volumes were calculated from regions-of-interest (ROI’s) drawn on all T_2_ anatomic image slices covering the tumor by an experienced murine pathologist (RB). These ROI’s were then overlaid on the corresponding HP metabolite images [1-^13^C] lactate, [1-^13^C] alanine, [1-^13^C] pyruvate, [^13^C] urea and ADC maps to derive mean whole tumor Lactate/Pyruvate (Lac/Pyr), Alanine/Pyruvate (Ala/Pyr) and Urea/normalized to kidney Urea values, as well as ADC values. Only voxels with metabolite signal-to-noise ratios ≥ 3 were considered in the analyses. Pixels on the boundary of the prostate or prostate tumor were not considered in order to reduce partial volume effects, utilizing only voxels that were 75% within the tumor.

### 2.7. Statistical Analysis

Data were presented as mean ± standard error of the mean (SEM). Statistical analysis was performed using PRISM 7 (GraphPad, La Jolla, CA, USA). One-way ANOVA was used with Tukey’s post hoc analysis for multiple comparisons. All *p*-values reported are corrected for multiple comparisons with an α = 0.05; * denotes *p* < 0.05, ** for *p* < 0.005, *** for *p* < 0.0005 and **** for *p* < 0.0001.

## 3. Results

### 3.1. Anatomical Changes with TRAMP Tumor Evolution and Progression

**Figure 3:**
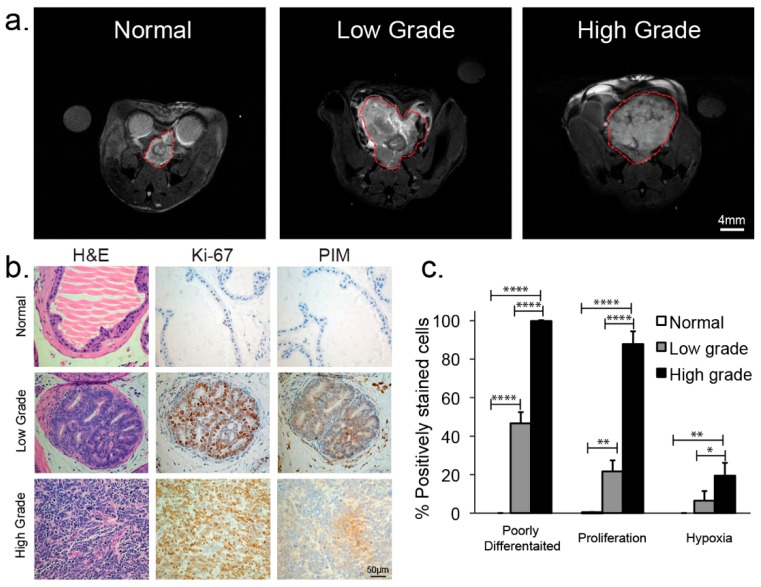
(**a**) Representative T_2_-weighted anatomic images of the normal murine prostate, low-grade and high-grade prostate cancer. (**b**) Immunochemical staining of excised representative normal murine prostate and low- and high-grade transgenic mouse model of prostate cancer (TRAMP) tumors; H&E section, Ki-67 staining, and Pimonidazole (PIM) staining (200× magnification). (**c**) Bar graph summarizing the mean ± sdev % positively poorly differentiated, Ki-67, and PIM stained cells of excised representative normal murine prostate and low- and high-grade TRAMP tumors. * denotes *p* < 0.05, ** for *p* < 0.005, **** for *p* < 0.0001.

Representative T_2_ weighted spin-echo images (Figure 3a) demonstrated both the T_2_-weighted contrast and size difference when progressing from the normal prostate (0.04 ± 0.01 cc) to low- (1.20 ± 0.6 cc) and high-grade tumors (3.39 ± 0.80 cc). Normal murine prostate histology was highly glandular with normal secretory epithelial cells lining glands and stromal tissue supporting the glands (Figure 3b). Low-grade TRAMP tumors demonstrated a loss of simple glandular morphology with the acini being filled by increased numbers of transformed epithelial cells and tumors composed primarily of well-differentiated and moderately well-differentiated malignant cells (Figure 3b,c). In contrast, high-grade tumors exhibited sheets of poorly-differentiated cells. Correspondingly, there was an overall decrease in T_2_ signal intensity in regions of low-grade disease as well as regions of higher T_2_ signal intensity associated with residual normal prostate tissue. In high-grade tumors, the entire tumor was associated with low T_2_ signal intensity (Figure 3a).

**Figure 4:**
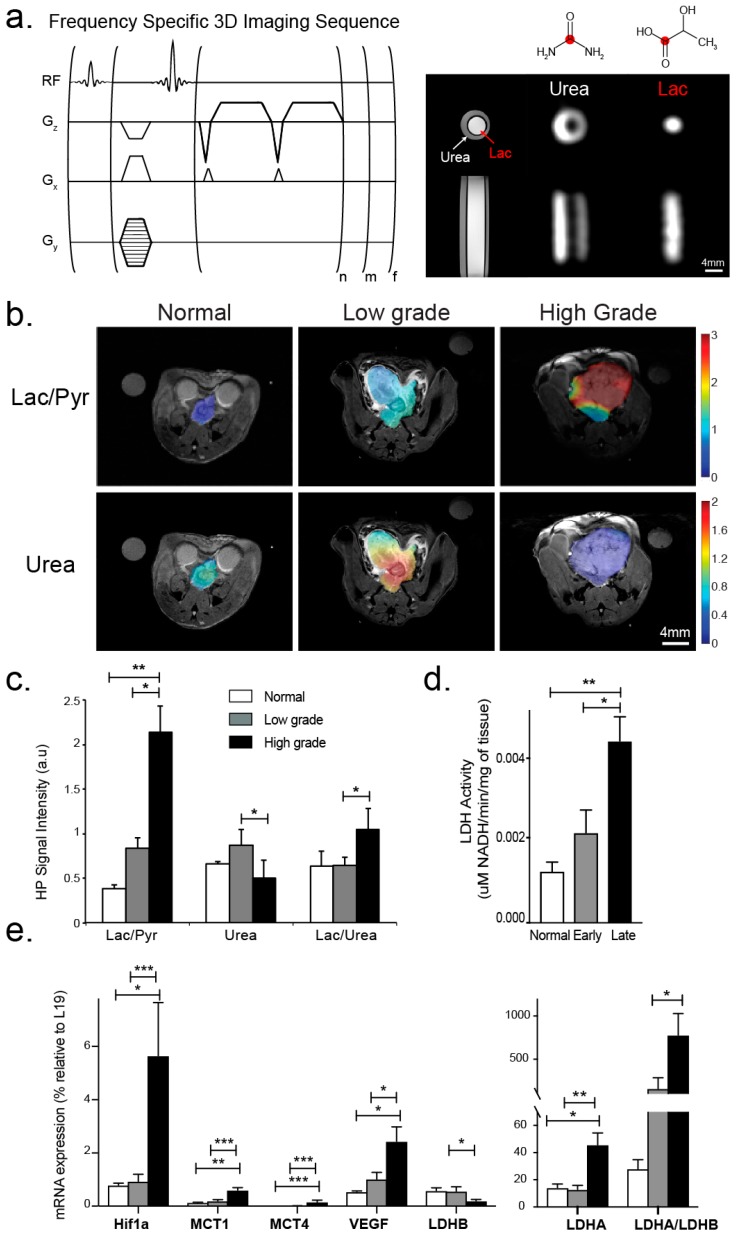
(**a**) (top, left) Pulse sequence diagram of the high-spatial resolution 3D GRASE HP ^13^C MRSI imaging approach used for in vivo HP ^13^C imaging in this study (n and m number of phase encodes and f is the number of frequencies). (**a**) (top, right) Resonances of [1-^13^C] lactate, [1-^13^C] alanine [1-^13^C] pyruvate, and [^13^C] urea were excited and imaged sequentially as shown for the ^13^C lactate and ^13^C urea phantom. (**b**) Representative HP ^13^C Lac/Pyr and ^13^C-urea images overlaid on the corresponding T_2_-weighted anatomic images for the normal prostate, and low- and high-grade cancer. The color scale of the images represents the magnitude of the Lac/Pyr ratio. (**c**) Bar graph showing mean ± sdev values for Lac/Pyr, Urea/kidney Urea and Lac/Urea ratio from the normal mouse prostates and low and grade tumors studied. (**d**) Corresponding mean ± sdev LDH activity values. (**e**) Corresponding mean ± sdev mRNA expression values of key transporters and enzymes associated with pyruvate and lactate transport and metabolism (*Mct1* and *Mct4*, *Ldha* and *Ldhb*) and of factors impacted by the hypoxic tumor microenvironment (*Hif1α* and *Vegf*). * denotes *p* < 0.05, ** for *p* < 0.005, *** for *p* < 0.0005.

While normal prostate was non-proliferative and normoxic, there was a significant progression in both proliferation (Figure 3c, Ki-67) and hypoxia (Figure 3c, PIM) from normal to low-grade and between low- and high-grade TRAMP tumors. As shown in Figure 4b,c, cells in high-grade tumors were almost all rapidly proliferating (88 ± 7% of tumor positive for Ki-67) and 19 ± 6% of the tumor was hypoxic (positive for PIM) as compared to low-grade tumors (22 ± 6% positive for Ki-67, 7 ± 5% for PIM) and normal TRAMP prostates (0.3 ± 0.2% positive for Ki-67, 0% positive for PIM).

The apparent water diffusion coefficient significantly decreased with the development of prostate cancer, from 1.5 ± 0.2 × 10^−3^ mm^2^/s in the normal mouse prostate to 1.1 ± 0.2 × 10^−3^ mm^2^/s in low-grade cancer (*p* < 0.05), and to 0.88 ± 0.16 × 10^−3^ mm^2^/s in high-grade cancer (*p* < 0.005) (Figure 2). In the normal prostate, the water ADC had close to a Gaussian distribution, meanwhile in low-grade disease, there was a non-Gaussian ADC distribution consistent with regions of normal and malignant ADC values, and consistent with the histopathological findings. In high-grade tumors most of the ADC values were below 1 × 10^−3^ mm^2^/s, consistent with the histopathological findings that the tumor primarily consisted of sheets of poorly-differentiated cancer cells with a complete loss of acinar morphology.

### 3.2. Changes in Perfusion and Lactate Metabolism with Prostate Cancer Evolution and Progression

High spatial resolution HP ^13^C MRSI data were acquired using the 3D GRASE approach (Figure 4a) from four normal murine prostates, nine low-grade TRAMP tumors and eleven high-grade TRAMP tumors, 35s after an injection of co-polarized [1-^13^C] pyruvate and ^13^C-urea. Figure 4b shows representative HP ^13^C Lac/Pyr and ^13^C-urea images overlaid on the corresponding T_2_-weighted anatomic images for the normal murine prostate, and low- and high-grade cancer. Hyperpolarized Lac/Pyr ratio increased with the development of prostate cancer, from 0.39 ± 0.04 in the normal prostate to 0.84 ± 0.12, and 2.16 ± 0.32 in low- and high-grade disease, respectively (Figure 4c). While the increase in the Lac/Pyr ratio between the normal prostate and low-grade cancer was non-significant (*p* = 0.2), there was a significant (*p* < 0.0001) 5.5-fold increase in HP Lac/Pyr for high-grade disease relative to normal, and a significant (*p* < 0.0005) 2.4 fold increase between low- and high-grade cancer. There was a non-significant increase in HP ^13^C urea/renal urea in low-grade cancer (0.87 ± 0.18) relative to the normal prostate (0.66 ± 0.03) with perfusion significantly (*p* < 0.05) decreasing in high-grade cancer (0.50 ± 0.20). The Lac/Urea ratio did not change between normal and low-grade cancer, but significantly increased 1.8 fold between low- and high-grade cancer (Figure 4c). The increase in HP Lac/Pyr ratio mirrored changes in LDH activity, with LDH activity non-significantly (*p* = 0.2) increasing 1.6 fold between normal and low-grade cancer, and then significantly (*p* < 0.005) increasing 3.4 fold in high-grade cancer vs. normal and 2-fold (*p* < 0.05) vs. low-grade cancer (Figure 4d).

Figure 4e summarizes the expression of key transporters and enzymes associated with pyruvate transport and metabolism (*Mct1* and *Mct4*, *Ldha* and *Ldhb*) and of factors impacted by the hypoxic tumor microenvironment (*Hif1α* and *Vegf*). Interestingly, there was no significant change in expression for any of the genes investigated between normal and low-grade prostate cancer. A key finding of this study was that all of the genes studied were significantly elevated between low- and high-grade prostate cancer. *Mct1* and *Mct4* were significantly up-regulated (*Mct1*: 3-fold, *p* < 0.005, *Mct4*: 20-fold, *p* < 0.0001) in high- versus low-grade TRAMP tumors. *Ldha* was significantly increased ≈3.5-fold (*p* < 0.005) and *Ldhb* significantly decreased by 3-fold (*p* < 0.005), which led to a dramatic 5-fold increase (*p* < 0.0001) in the *Ldha/Ldhb* ratio in high- versus low-grade TRAMP tumors. Due to increased hypoxia in the tumor microenvironment (higher PIM staining), there was also a significant 12-fold (*p* < 0.0005) and 2.5-fold (*p* < 0.05) increase in *Hif1α* and *Vegf* expression, respectively, in high- versus low-grade TRAMP tumors. In both low- and high-grade cancers we observed significant correlations between *Hif1α* expression with *Ldha* (R = 0.97 for both), *Mct4* (R = 0.99 and 0.94) and *Vegf* (R = 0.80 and 0.98). There was also a good correlation between the Lac/Urea ratio and the *Hif1α* and *Vegf* mRNA expression (R = 0.90 and 0.83, respectively).

### 3.3. Changes in Lactate Metabolism with Ldha-Knockout

**Figure 5:**
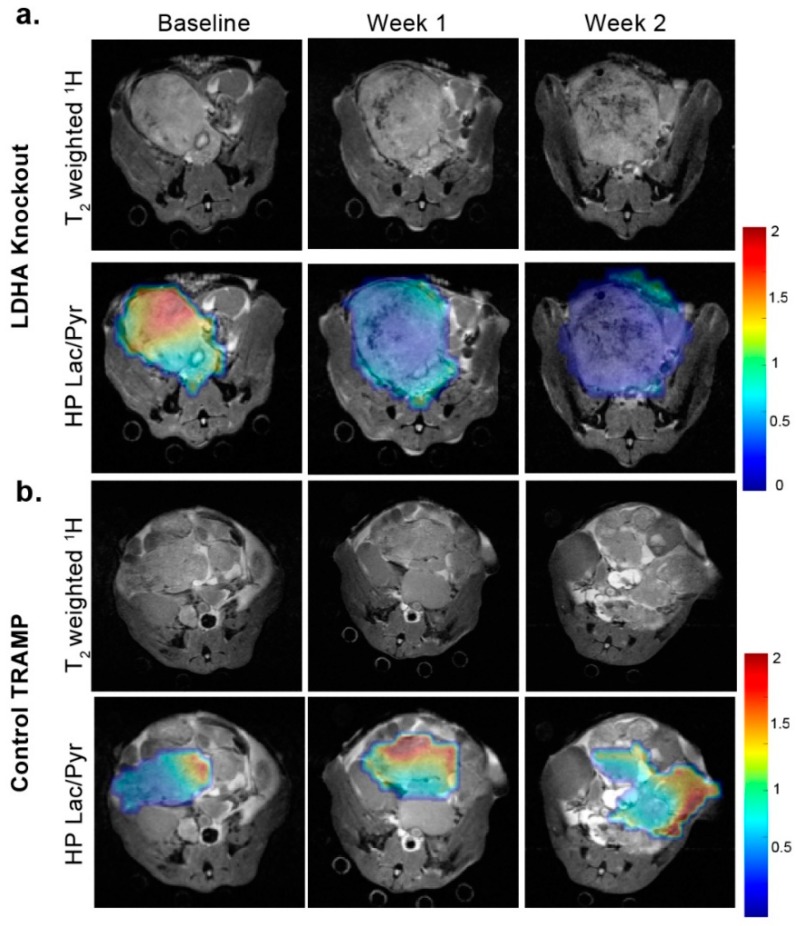
Representative T_2_^−^ weighted and overlaid HP Lac/Pyr images from an LDHA-TRAMP knockout mouse (**a**) and a vehicle control LDHA-TRAMP mouse (**b**), at baseline, 1-week and 2-weeks following the administration of tamoxifen (LDHA-knockout), or vehicle control (LDHA-intact). The color scale of the images represents the magnitude of the Lac/Pyr ratio. Scale bar: 0–2 relative Lac/Pyr ratio.

**Figure 6:**
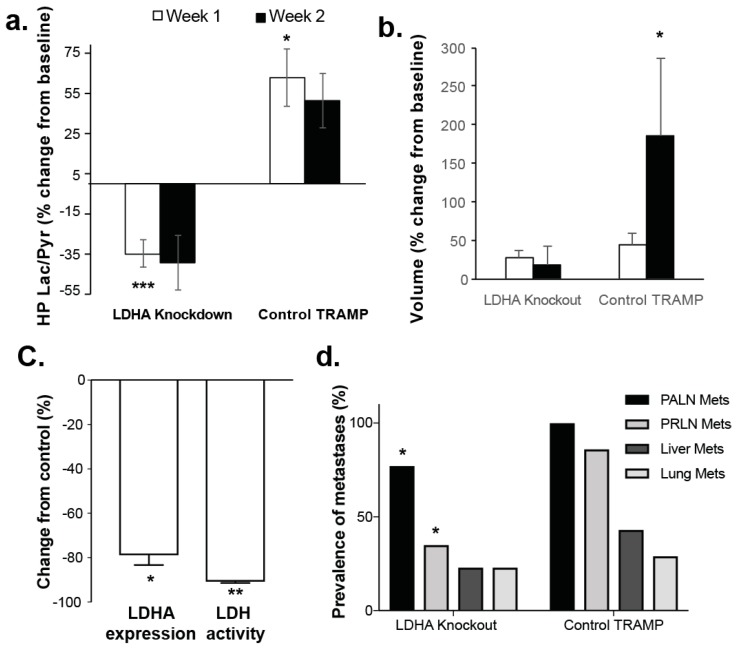
(**a**) Graphical presentation showing the mean ± sdev % changes in the HP Lac/Pyr from baseline of tamoxifen (LDHA-knockout), or vehicle control (LDHA-intact). (**b**) Corresponding mean ± sdev % volume changes from baseline. (**c**) Mean ± sdev % change from control of in *Ldha* expression and LDH activity in *Ldha*-knockout mice. (**d**)% prevalence of metastasis in control TRAMP to that in *Ldha*-knockout mice, concentrating on several typical sites of disease spread: regional lymph nodes (PALN), the more distant lymph nodes (PRLN), and visceral metastases (liver and lung). * denotes *p* < 0.05, ** for *p* < 0.005, *** for *p* < 0.0005.

A total of 26 LDHA-TRAMP mice were utilized in the *Ldha*-knockout studies: 18 of which had *Ldha*-knockout accomplished by administration of tamoxifen while the remaining 8 were administered only vehicle to serve as controls. Figure 5 shows representative T_2_-weighted and overlaid HP Lac/Pyr images from an LDHA-TRAMP knockout mouse (Figure 5a) and a vehicle control LDHA-TRAMP mouse (Figure 5b), at baseline, 1-week and 2-week time-points following the administration of tamoxifen (*Ldha*-knockout) or vehicle control (*Ldha*-intact). In the *Ldha*-knockout TRAMP there was a visually clear decrease in the HP Lac/Pyr ratio by 1 week that further decreased by 2 weeks. The control TRAMP, in contrast, demonstrated an increase in the HP Lac/Pyr ratio by 1 week that remained relatively constant by 2 weeks (Figure 5a). As shown quantitatively in Figure 6a, there was a significant (*p* < 0.0005) reduction in Lac/Pyr ratio at 1 and 2 weeks in *Ldha*-knockout mice, while in controls there was a mean significant (*p* < 0.05) increase in the Lac/Pyr ratio at 1 week that did not change by the 2 week time point. Tumor volume did not significantly change from baseline for the *Ldha*-knockout mice but significantly (*p* < 0.05) increased for the control mice during the same time frame (Figure 6b). Reduction in the Lac/Pyr ratio correlated with both a significant reduction in *Ldha* expression (79 ± 6%, *p* < 0.05) and LDH activity (85 ± 3%, *p* < 0.005) (Figure 6c). There was no change in the expression of the monocarboxylate transporters (*Mct1* and *Mct4*) in *Ldha*-knockout tumors, nor was there a change in HP ^13^C urea perfusion and expression of factors impacted by the hypoxic tumor microenvironment (*Hif1α* and *Vegf*). Similar to the tumor volume, the mean ADC did not change significantly by week 1 (*p* = 0.08) after *Ldha*-knockout and remained only slightly higher than baseline at week 2 (10%, *p* = 0.23).

We also carried out an assessment of metastatic disease, comparing the incidence of metastasis in control TRAMP (*Ldha*-intact) to that in *Ldha*-knockout TRAMP, concentrating on several typical sites of disease spread: regional periaortic lymph nodes (PALN), the more distant perirenal lymph nodes (PRLN), and visceral metastases (liver and lung). As expected, the incidences of metastases were highest in the regional lymph nodes (100% and 77%, respectively) and progressively lower in more distant tissues (43% and 23% in liver, respectively). We observed a reduction in metastases at all of the sites investigated in *Ldha*-knockout mice relative to controls; with a significant (*p* < 0.05) reduction in both the regional (PALN) and more distant (PRLN) lymph nodes (Figure 6d).

## 4. Discussion

In this study we used a high magnetic field (14T), high-spatial resolution, dual-agent (^13^C-pyruvate and ^13^C-urea) HP ^13^C and multi-parametric (T_2_ and DWI) ^1^H MRI approach to study changes in prostate cancer perfusion and the Warburg effect with progression from the normal murine prostate to low- and high-grade primary prostate cancer and metastases using the TRAMP model. At 14T, it was possible to image anatomy, water diffusion, perfusion and metabolism in the normal murine prostate, which is only ≈ 4mm in diameter, as well as in small volume low-grade disease, which demonstrated regions of cancer and benign prostate tissue as is observed in the human situation. This was not possible in prior pre-clinical studies using a 3T MRI scanner [22]. T_2_-weighted anatomical and diffusion-weighted MRI are the common components of multi-parametric ^1^H MRI prostate cancer patient exams. Similar to human prostate cancer [27], TRAMP tumors demonstrated a decrease in T_2_ signal intensity during prostate cancer development. This reduction in T_2_-weighted MRI image signal intensity is due to a loss of the normal glandular (ductal) morphology that occurs in regions of prostate cancer in patients as well as in the TRAMP model [27]. 

Diffusion weighted imaging (DWI) is sensitive to the motion of water molecules at microscopic spatial scales within biological tissues, and the apparent water diffusion coefficient (ADC) can provide unique information about microscopic tissue compartments, as well as pathology of prostate tissues [28]. In patients, the ADC has been shown to be lower in prostate cancer than in surrounding benign prostate tissues, with typical ADC values ranging from 2.0 to 1.4 and 1.6 to 0.8 × 10^−3^ mm^2^/s, respectively [29,30]. DWI has also shown promise for reflecting the pathologic grade of prostate cancer, with lower ADC values found in higher Gleason Grade cancers; ADC decreased from 1.135 ± 0.119 to 0.976 ± 0.103 to 0.831 ± 0.087 mm^2^/s from patients with Gleason score 3 + 3, 3 + 4, and 4 + 3 cancers, respectively [31]. This trend is recapitulated in the TRAMP model, with ADC significantly decreasing from 1.5 ± 0.2 × 10^−3^ mm^2^/s in the normal mouse prostate to 1.1 ± 0.2 × 10^−3^ mm^2^/s in low-grade cancer, and to 0.88 ± 0.16 × 10^−3^ mm^2^/s in high-grade cancer. Pathologically, there was a progression from open glandular structures, which harbor large regions of free moving fluid in the normal prostate, to packed sheets of dedifferentiated cells in high-grade cancer, which resulted in a significant change in water diffusion and coincided with the measured change in ADC. Interestingly, ADC values of low-grade TRAMP tumors demonstrated a high variance with punctuated regions of both normal ADCs as well as regions of reduced ADC corresponding to areas of cancer with surrounding benign prostate tissues as is typically observed in prostate cancer patients.

An important finding of this study was the significant increase in the Warburg effect in high-grade prostate cancer. The Warburg effect, as measured by the tumor HP Lac/Pyr ratio (Figure 4c) and LDH activity (Figure 4d), went up in low-grade prostate cancer relative to the normal prostate, although this increase was not significant. However, there was a large and significant increase in the HP Lac/Pyr ratio (Figure 4c) and LDH activity (Figure 4d) in high- versus low-grade disease and the normal prostate. This finding suggested that the measurement of the HP Lac/Pyr ratio may provide a means of discriminating high- from low-grade disease at the time of biopsy diagnosis of prostate cancer, thereby helping to reduce over-treatment of low-risk disease but directing those with aggressive disease to curative treatment [2,3]. The increase in HP Lac/Pyr in prostate cancer in this pre-clinical study is consistent with prior publications investigating the steady lactate concentration in prostate cancer patient biopsies [32], HP ^13^C MRSI studies of the TRAMP model [22,24], and a HP ^13^C MRSI study of patient derived living prostate tissue slices [9]. The 2.4-fold increase in the HP Lac/Pyr ratio between low- and high-grade cancer observed in this study mirrored the 2.9-fold increase in HP [1-^13^C] pyruvate to HP [1-^13^C] lactate flux (*k_PL_*) between low- and high-grade cancer observed in a prior 3T HP ^13^C MRSI study using the TRAMP model [22]. However, a comparison between normal murine prostate and low-grade disease was not made in this prior publication and an important new finding of this study was the lack of significant increase in the HP Lac/Pyr ratio (i.e., the Warburg effect) in low-grade prostate cancer versus normal tissue, with significance only occurring with progression to aggressive, high-grade disease. This finding is consistent with prior publications suggesting that the Warburg effect does not become important in prostate cancer until late-stage/high-grade disease [33,34,35,36]. These publications argue that early-stage/low-grade prostate cancers rely on lipids and other biologic fuels for energy production [33,34,35,36], and that the glycolytic phenotype is only associated with the evolution of aggressive disease [37,38].

Another important finding of this study was the importance of the hypoxic tumor microenvironment in driving the elevated Warburg effect in high-grade prostate cancer. Tumor perfusion based on HP ^13^C-urea increased slightly in low-grade cancer relative to the normal prostate, but significantly decreased with high-grade disease. This resulted in a significantly higher level of hypoxia in high-grade prostate cancer based on PIM staining. The oxygen-sensitive HIF1α transcription factor has been found to be up-regulated in regions of tumor hypoxia and increases the expression of angiogenesis factors such as VEGF to increase oxygen delivery as well as increasing aerobic glycolysis through increasing the expression and activity of key enzymes in the glycolytic pathway, such as LDHA [8]. Consistent with this scenario, a significant increase was observed in the mRNA expression of *Hif1α*, *Vegf*, and *Ldha*, as well as an increase in LDH activity in high- versus low-grade TRAMP tumors, while no change was seen in the expression of these enzymes between normal and low-grade disease. Hypoxic prostate cancers, which induce *HIF1α* and glycolysis most strongly, tend to be of higher Gleason grade, are more invasive and metastatic, and less responsive to therapy than those with normal oxygen levels [39,40]. Moreover, *Ldhb* significantly decreased and the *Ldha/Ldhb* ratio significantly increased in high-versus low-grade prostate cancer while there was no significant difference in either the *Ldhb* or *Ldha/Ldhb* ratio between low-grade cancer and the normal prostate. This is consistent with a recent paper reporting that prostate cancer expressed higher levels of *Ldha* and phosphorylated *Ldha*, and lower levels of *Ldhb*, than normal tissue, and that a high *Ldha/Ldhb* ratio was a marker of poor clinical prognosis [41].

Additionally, high-grade disease in this murine study was associated with significantly increased expression of the monocarboxylate transporters *Mct1* and *Mct4* that are involved in pyruvate and lactate transport in and out of the tumor [8,9]. The significant increase in *Mct4* observed in high-grade TRAMP tumors resulted in increased export of lactate out of the cells, which is important for reducing intracellular lactate levels via lactate-H^+^ co-export, thereby maintaining a physiologic intracellular pH and allowing a high metabolic flux through the LDH enzymatic reaction [34,42]. There was no increase in the expression of *Mct1* and *Mct4* transporters between low-grade disease and normal prostate tissue. A more avidly *Mct1* and *Mct4* expressing phenotype has been correlated with a more aggressive and therapeutically resistant prostate cancer [37,38].

Tumor excretion of lactic acid, combined with poor tumor perfusion, results in an acidic extracellular pH in tumors compared with normal tissue [4,10]. This acidification of the tumor microenvironment has also been shown to occur in the TRAMP model [21,43]. The resulting acidic environment promotes cancer aggressiveness and metastasis by facilitating a degradation of the extracellular matrix by proteinases [11,12], increasing angiogenesis through the release of VEGF [44], and inhibiting the immune response to tumor antigens [14]. Extracellular acidification also may render prostate tumors chemo- and radiation-resistant [14,45]. Taken together, these observations suggest that not only increased lactic acid production, but also its efflux are important parameters associated with aggressive prostate cancer [8,46]. Moreover, tumor-specific metabolic shifts, such as increased production and efflux of lactate, can potentially be exploited for cancer therapy with minimal impact on normal tissues [10].

Another important finding of this study was the demonstration of the functional importance of increased lactate production and efflux in the progression of primary prostate cancer and its metastases. In this study, it was demonstrated that the genetic knockdown of *Ldha* in prostate tumors could be monitored via the rate of conversion of HP ^13^C-pyruvate to ^13^C-lactate, with a significant reduction in HP Lac/Pyr in *Ldha*-knockdown mice versus a significant increase in control mice occurring by the 1-week timepoint. The reduction in HP Lac/Pyr in *Ldha*-knockdown mice correlated with an approximate 80% reduction in *Ldha* expression and LDH activity, but no changes in the monocarboxylate transporters (*Mct1* and *Mct4*), nor in HP ^13^C urea perfusion or expression of factors impacted by the hypoxic tumor microenvironment (*Hifα* and *Vegf*), thus demonstrating the specificity of *Ldha*-knockdown. More importantly, *Ldha* gene knockdown had salient functional effects on prostate tumors in this transgenic model, including significantly reduced primary tumor growth and significantly reduced lymph node and visceral metastases. Interestingly, these preliminary studies indicated that HP ^13^C biomarkers could detect early, metabolic shifts indicative of tumor regression, before classical morphometric parameters (i.e., tumor volume & ADC) change. Others have demonstrated previously the therapeutic effect of *Ldha*-knockdown in other tumor types in mice, including renal cell cancer, hereditary leiomyomatosis [16], neuroblastoma [47], breast cancer [48,49] and lung cancer [50]. This is the first report of such effect in prostate tumors in vivo.

*Ldha* expression levels have been correlated previously with metastasis and poor prognosis in several tumor types [6]. The exact mechanisms are not fully understood, but as described above decreased interstitial pH in the tumor microenvironment, probably largely as a consequence of lactate production by tumors, is thought to increase activation of extracellular proteases like matrix metalloproteinases and cathepsins [51] and to reduce immunologic responses, in part through up-regulation of PD-L1 [15,52,53]. The fact that the incidence of metastases is not eliminated entirely in this study suggests either that knockdown of *Ldha*/LDH-5 activity was initiated in most animals after tumors had advanced to the state where cancer cell dissemination was occurring, or that certain tumors/tumor cells compensate for the loss of *Ldha* and, after a lag period, are capable of undertaking invasion and metastasis.

One limitation of this study was that TRAMP tumors are spatially heterogeneous with respect to hypoxia and most likely it is only hypoxic regions of the tumor microenvironment that contribute to increased glycolysis as measured by the hyperpolarized Lac/Pyr ratio. Unfortunately, it was not technically feasible to accurately register the different tumor regions of upregulated glycolysis on imaging to the corresponding hypoxic tissue regions visible at PIM IHC after tumor dissection. Fortunately, there was a large significant difference in the mean amount of PIM staining (hypoxia) between normal murine prostate and low-grade tumors as compared to high-grade tumors in this study (Figure 3c) thereby allowing conclusions to be drawn about the role of the hypoxic tumor microenvironment in driving the glycolytic shift in high-grade prostate cancer. The correlation between hypoxia and the Lac/Pyr ratio can be directly correlated in vivo by performing a combination of EPR imaging of the trityl radical, used for hyperpolarization, to measure pO_2_ and hyperpolarized [1-^13^C] pyruvate MRSI of the same murine tumor [54], however, this is beyond the scope of this manuscript.

Another limitation of this study, as with most pre-clinical murine studies, is how well the Warburg effect in the TRAMP model recapitulates the human situation. Therefore, it will be necessary to establish in patient studies the important role of the Warburg effect as a marker of prostate cancer aggressiveness and as a therapeutic target as has been shown in this pre-clinical study. A number of factors support the feasibility of clinically translating the findings of this pre-clinical study to accomplish these goals. Hyperpolarized [1-^13^C] pyruvate is already FDA IND approved for use in prostate cancer patients [55], and the clinical translation of HP ^13^C-urea and its co-polarization with [1-^13^C] pyruvate for combined metabolic and perfusion imaging after a single injection of hyperpolarized probes has been NIH-funded and is in progress. Hyperpolarized ^13^C-pyruvate MRSI has been added to a clinical multi-parametric ^1^H MRI prostate cancer staging exam, and initial prostate cancer patient studies have shown the ability to acquire both high-spatial (0.5 cc) and temporal (2 s) resolution 3D dynamic HP ^13^C MRSI data from throughout the human prostate in 47 seconds using an echo-planar spectroscopic imaging (EPSI) sequence on a clinical 3T MRI [56]. Based in part on the results of this pre-clinical study, patient studies have been initiated to investigate the relationship between the rate of conversion of hyperpolarized lactate and cancer aggressiveness (NCT02526368) and response to therapy (NCT02911467). Ultimately, hyperpolarized ^13^C MRSI may provide a highly personalized approach for prostate cancer therapy by: (i) stratifying patients into those who would benefit most from tumor glycolysis inhibition; (ii) enabling early determination of hitting the therapeutic target (*Ldha* inhibition/LDHA activity), and (iii) providing a better prediction of treatment response.

## 5. Conclusions

Consistent with prior publications [22,24] the progression from low- to high-grade prostate cancer in the TRAMP model resulted in a significant increase in the HP Lac/Pyr ratio (i.e., the Warburg effect). An important new finding of this study was the lack of a significant increase in the HP Lac/Pyr ratio (i.e., the Warburg effect) in low-grade prostate cancer versus normal tissue, suggesting that HP Lac/Pyr may provide a sensitive marker of whether individual patients have aggressive disease at the time of biopsy diagnosis, a critical time for making decisions about whether active surveillances are an appropriate management approach or whether immediate aggressive treatment is required. Another important finding of this study was the importance of the hypoxic tumor microenvironment in driving the elevated Warburg effect in high-grade prostate cancer. More importantly, this study demonstrated that increased LDH activity and HP Lac/Pyr played an important role in tumor progression and metastases in this preclinical model of prostate cancer. These preclinical findings provide motivation for patient studies investigating drugs inhibiting LDHA activity alone or in combination with other prostate cancer treatments.

## Supplementary Materials

The following are available online at https://www.mdpi.com/2072-6694/11/2/257/s1, Figure S1: Western blot of LDHA gene knockdown induced by tamoxifen administration.
