# Supplementary material for: The Role of Lactate Metabolism in Prostate Cancer Progression and Metastases Revealed by Dual-Agent Hyperpolarized 13C MRSI
Source: Cancers (Basel). 2019 Feb 22;11(2):257. doi: 10.3390/cancers11020257 (PMC6406929; doi:10.3390/cancers11020257)
Supplement: Supplementary file 1 [file cancers-11-00257-s001.pdf]

Supplementary Materials: The Role of Lactate Metabolism in Prostate Cancer Progression and Metastases Revealed by Dual-Agent Hyperpolarized ^{13}C MRI

Robert Bok, Jessie Lee, Renuka Sriram, Kayvan Keshari, Subramaniam Sukumar, Saeed Daneshmandi, David E. Korenchan, Robert R. Flavell, Daniel B. Vigneron, John Kurhanewicz and Pankaj Seth

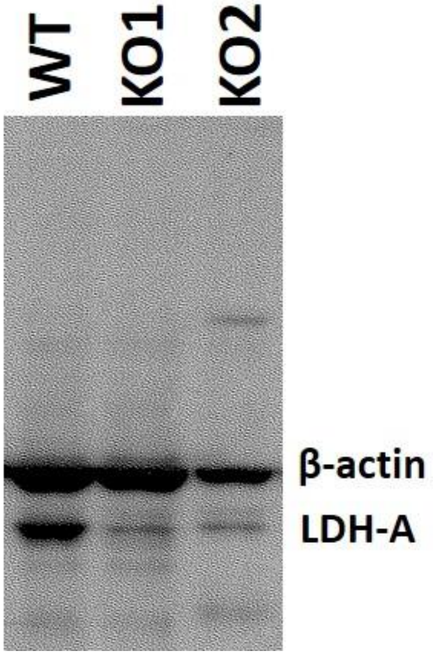

Figure S1. Western blot of LDHA gene knockdown induced by tamoxifen administration. B-actin was used as control.

© 2019 by the authors. Submitted for possible open access publication under the terms and conditions of the Creative Commons Attribution (CC BY) license (http://creativecommons.org/licenses/by/4.0/).
